# 

**Published:** 1887-12

**Authors:** 


WHOLE NUMBER CCCXXIX

DECEMBER, 1887.

NUMBER 5.
VOLUME XXVII.

### THE BUFFALO Medical AND Surgical Journal

ESTABLISHED YEAR 1844.

**EDITORS:**

**THOS. LOTHROP, M. D.** **A. R. DAVIDSON, M. D.**

ASSOCIATE EDITORS:

FLOYD S. CREGO, M. D. CARLTON C. FREDERICK, M. D.
FRED. R. CAMPBELL, M. D. FRANK H. POTTER, M. D.

EDWARD B. ANGELL, M. D., Rochester. N. Y.

Entered at the Post-Office at Buffalo, N. Y., as Second-class Mail Matter.

